# Deep learning‐mediated prediction of concealed accessory pathway based on sinus rhythmic electrocardiograms

**DOI:** 10.1111/anec.13072

**Published:** 2023-08-02

**Authors:** Lei Wang, Fang Yang, Xiao‐Jing Bao, Xiao‐Ping Bo, Shipeng Dang, Ru‐Xing Wang, Feng Pan

**Affiliations:** ^1^ Department of Cardiology The Affiliated Wuxi People's Hospital of Nanjing Medical University Wuxi China; ^2^ Key Laboratory of Advanced Process Control for Light Industry (Ministry of Education) Jiangnan University Wuxi China

**Keywords:** concealed accessory pathway, convolution neural network, deep learning, electrocardiograms, prediction

## Abstract

**Background:**

Concealed accessory pathway (AP) may cause atrial ventricular reentrant tachycardia impacting the health of patients. However, it is asymptomatic and undetectable during sinus rhythm.

**Methods:**

To detect concealed AP with electrocardiography (ECG) images, we collected normal sinus rhythmic ECG images of concealed AP patients and healthy subjects. All ECG images were randomly allocated to the training and testing datasets, and were used to train and test six popular convolutional neural networks from ImageNet pre‐training and random initialization, respectively.

**Results:**

We screened 152 ECG recordings in concealed AP group and 600 ECG recordings in control group. There were no statistically significant differences in ECG characteristics between control group and concealed AP group in terms of PR interval and QRS interval. However, the QT interval and QTc were slightly higher in control group than in concealed AP group. In the testing set, ResNet26, SE‐ResNet50, MobileNetV3_large_100, and DenseNet169 achieved a sensitivity rate more than 87.0% with a specificity rate above 98.0%. And models trained from random initialization showed similar performance and convergence with models trained from ImageNet pre‐training.

**Conclusion:**

Our study suggests that deep learning could be an effective way to predict concealed AP with normal sinus rhythmic ECG images. And our results might encourage people to rethink the possibility of training from random initialization on ECG image tasks.

## Graphical abstract

In the present study, six state‐of‐art convolutional neural networks models were trained with electrocardiography (ECG) images of concealed accessory pathway (AP) patients and control patients. This study provided a low‐cost, rapid, and noninvasive method to diagnose patients with concealed AP with normal sinus rhythmic ECG images. And adopting models trained from random initialization may be a good choice when we deal with small medical dataset.

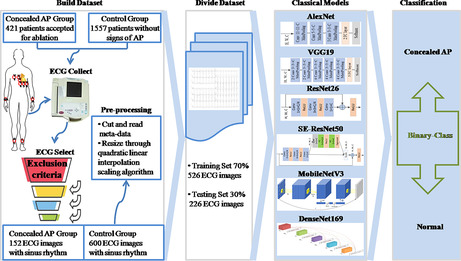

## 1 INTRODUCTION

Accessory pathway (AP), located between atria and ventricles, generates an anatomic circuit mediating macroreentrant tachycardia, leading to palpitations of patients and decline in life quality (Bagliani et al., 2020). Most of APs can conduct both anterogradely and retrogradely, while some APs are capable of propagating impulses in only one direction. When the AP is capable of anterograde conduction, ventricular pre‐excitation is commonly observed in patients with Wolf–Parkinson–White (WPW) syndrome, which is referred to as manifest AP and can be diagnosed by sinus rhythmic electrocardiography (ECG) with several signs, including delta wave, short PR interval, and broad QRS complex (Nikoo et al., 2022). On the contrary, APs that conduct only in the retrograde direction occur more frequently, referred to as concealed AP. The concealed APs cannot be diagnosed with ECG by cardiologists during sinus rhythm, but can be confirmed by the onset of tachycardia and electrophysiology procedure. Patients with concealed APs may go undiagnosed for years (Sacks et al., 2020). Therefore, it is important to develop a low‐cost and noninvasive method to detect concealed APs.

Electrocardiography is an easy and rapid tool for diagnosis of heart diseases. However, ECGs may contain crucial information that was not recognized even by well‐trained cardiologists (Goto et al., 2019). With the rapid development of artificial intelligence (AI) in computer vision and medical image, automatic identification of such subtle abnormalities in ECGs increases the rate of early diagnosis of arrhythmias with high accuracy. Considering convolutional neural networks (CNNs) has been shown outstanding performance in medical image analysis tasks in recent years because of its ability of preserving spatial relationships when filtering input medical images (Attia, Noseworthy, et al., 2019; Kaspal et al., 2021; Kwon et al., 2020). In contrast to handcrafted features, CNNs are able to automatically learn the most predictive features associated with heart failure, atrial fibrillation, and paroxysmal supraventricular tachycardia (PSVT) directly from 12‐lead ECG waveform based on the training samples (Attia, Kapa, et al., 2019; Attia, Noseworthy, et al., 2019; Jo et al., 2021; Ker et al., 2018). However, it is not yet unclear about the effectiveness of CNNs on identifying concealed AP with normal sinus rhythm ECGs. In addition, fine‐tuning the pre‐trained CNNs is a preferred strategy for small dataset according to conventional wisdom (Apostolopoulos & Mpesiana, 2020). However, before the prevalence of the “pre‐training and fine‐tuning” paradigm, classifiers were usually trained from scratch with no pre‐training, which is somewhat overlooked today when the target tasks have less training data.

The purpose of this study is to evaluate the effectiveness of state‐of‐the‐art CNNs on predicting concealed AP with normal sinus rhythm ECGs and the superior performance between randomly initialized models and fine‐tuning pre‐trained models. We presented experiments on these models, including AlexNet, VGG19, Resnet26, SE‐Resnet50, MobilenetV3_large_100, and DenseNet169, and also provided a detailed experimental analysis on the performance of the above models, in terms of accuracy, sensitivity, specificity, precision, F1 score, ROC curve, and area under the curve, to demonstrate the effectiveness and value of these selected CNNs.

## 2 METHODS

### 2.1 Study protocol

**FIGURE 1:**
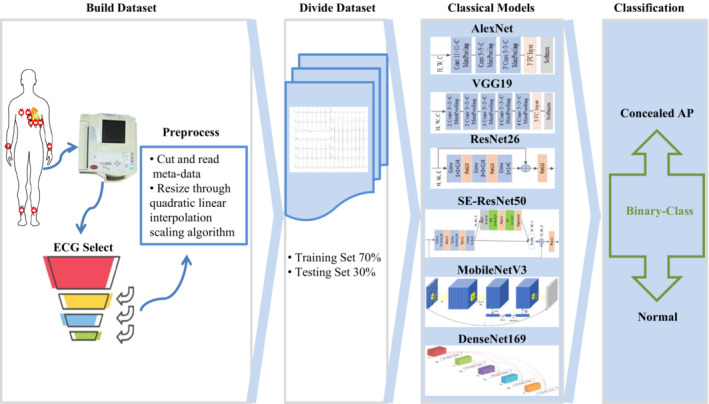
Schematic representation of the process of this study. The ECG images were selected and split into the training set and testing set in a 7:3 ratio. ECG images in the training set were used to train six state‐of‐art CNN models respectively. ECG images in the testing set were used to evaluate the screening performance of the selected CNN models; ECG, electrocardiography; CNN, convolutional neural networks.

The study methods and steps were data collection, ECG images preprocessing, dataset set‐up, selecting the state‐of‐art networks for training, and classifying ECG images of concealed AP and healthy subjects. A schematic representation of our proposed method is given in Figure 1.

### 2.2 ECG collection

Under the approval of the ethics committee of the affiliated Wuxi People's Hospital of Nanjing medical university, ECGs of patients with concealed AP were collected from January 1, 2013, to August 31, 2021, and ECGs from healthy subjects in control group were collected between January 1, 2020, and January 13, 2020. All ECG recordings in both control and concealed AP groups were digital, standard 10‐s, 12‐lead sinus rhythmic ECGs, sampled at 500 Hz using MAC 800 or 1200ST ECG machine (GE Healthcare). The bandwidth of filter setting was 0.16–40 Hz. Each ECG recording was collected and labeled by one cardiologist, and confirmed by another cardiologist with the label results. Also, two cardiologists would discuss the disagreement and draw a final conclusion.

### 2.3 ECG selection

**FIGURE 2:**
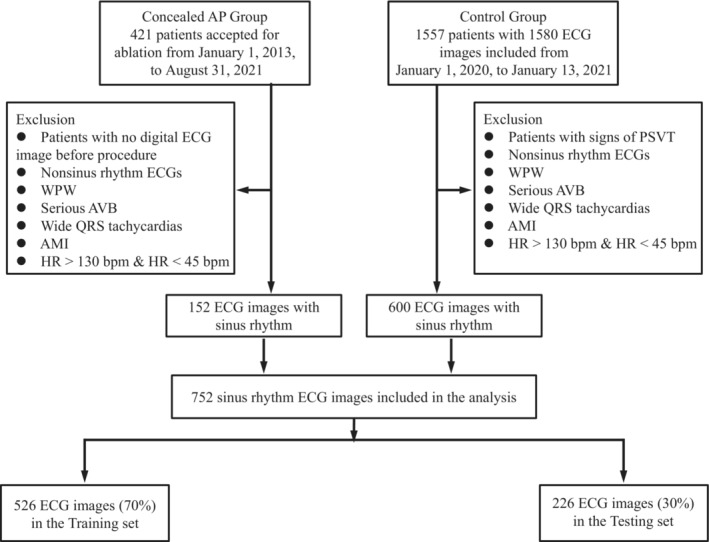
Flowchart of data collection and set creation. ECGs of 421 patients with concealed AP and 1557 control patients were also collected. After exclusion of nonsinus rhythm ECGs, WPW syndrome, serious AVB, wide QRS tachycardias, acute myocardial infarction, HR >130 bpm, and HR <45 bpm , a total of 752 ECGs (152 ECGs for concealed AP group and 600 ECGs for control group) were included in this study. These ECGs were randomly divided into two datasets, including training set (*n* = 526) and testing set (*n* = 226); ECG, electrocardiography; AP, accessory pathway; WPW, Wolf–Parkinson–White; HR, heart rhythm.

Patients in concealed AP group were diagnosed by electrophysiological study and radiofrequency ablation, and were included in the study only if they had a sinus rhythmic ECG before the procedure. Patients in the control group were evaluated without evidence of AP in the outpatient clinic by a cardiologist via history collection, medical records, or telephone follow‐up. The exclusion criteria were as follows: nonsinus rhythmic ECGs, WPW syndrome, serious atrioventricular bundle block, wide QRS tachycardia, acute myocardial infarction, heart rhythm (HR) >130 bpm, and HR <45 bpm for both groups (Figure 2). This study was carried out in accordance with The Code of Ethics of the World Medical Association (Declaration of Helsinki).

### 2.4 ECG preprocessing

It is worth mentioning that a complete ECG not only contains physiological signal waveform diagram but also contains some metadata, such as sex, age, heart rate, PR interval, and QT interval, which will interfere with the feature extraction of the model. Therefore, the metadata part of ECG recordings was cut out from original ECG images (resolution of 6786*4731) and the physiological signal waveform diagram was kept with a resolution of 6600*3300. And then, all ECG images were resized to 1600*800 as the input for the model through quadratic linear interpolation scaling algorithm, with the aim of retaining as much waveform information as possible to detect the subtle features.

### 2.5 Dataset set‐up

All ECG recordings were randomly split into training set and testing set at a ratio of 7:3. There was no overlap among these two sets as the ECG recordings from one patient can only be grouped into the same set. Six classical CNN models were trained through the training set and the remaining 30% ECGs in testing set were used to characterize the performance of the above six models for detecting the concealed AP.

### 2.6 The proposed CNNs

In the present study, a total of six well‐known CNN architectures (AlexNet, VGG19, Resnet26, SE‐Resnet50, MobilenetV3_large_100, and DenseNet169) were trained to distinguish concealed AP from standard 12‐lead ECGs acquired during normal sinus rhythm. We implemented the above networks on AI station with Linux Centos 7.8, and GPU of NVIDIA PCIe A100 (40 GB). All experiments were performed under Pytorch backend with Python, by utilizing Numpy, Matplotlib, and other deep learning libraries.

The network architectures of AlexNet, VGG19, ResNet26, SE‐ResNet50, MobileNetV3_large_100, and DenseNet169 were introduced as follows.

#### 2.6.1 AlexNet

AlexNet is the representative of deep neural networks, which was designed by Hinton et al. AlexNet mainly adopts three sizes of convolution kernel, 11 × 11, 5 × 5, and 3 × 3. Highlights in AlexNet include the use of ReLU as active function, the use of maxpooling, the use of dropout layer avoiding overfitting, the combination of two fully connected layers, and one softmax layer used to output the final result, which are often adopted as effective tricks for the subsequent CNNs (Krizhevsky et al., 2017).

#### 2.6.2 VGG19

VGGNet is another deep neural network with a big improvement after AlexNet. Compared with AlexNet, VGG only uses a small convolution kernel: 3 × 3, but deepens to 19 layers in terms of network depth. Stacking of small convolution kernel not only increases the nonlinearity of network, but also reduces the number of parameters (Simonyan & Zisserman, 2015).

#### 2.6.3 ResNet26

**FIGURE 3:**
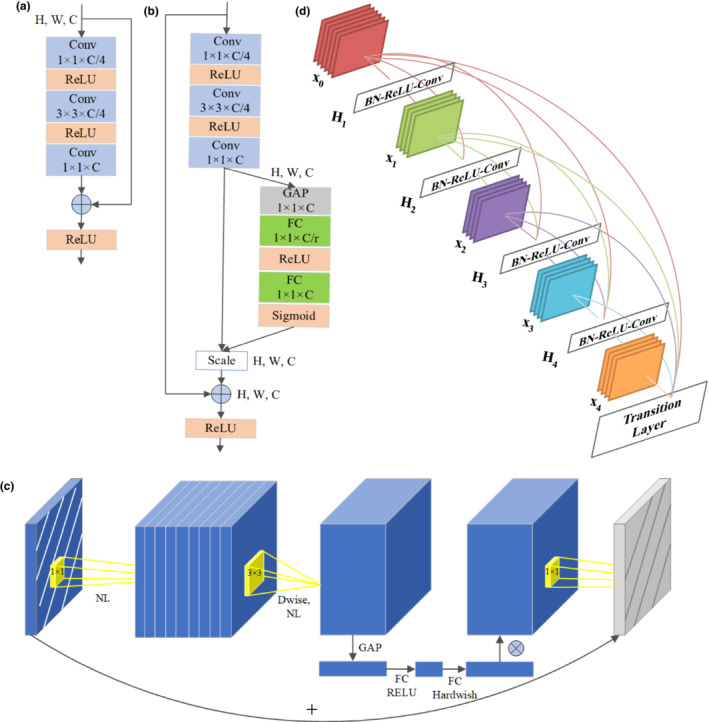
Architecture of the CNN models. (a) The architecture of bottleneck block. (b) The schema of SE‐ResNet bottleneck block. (c) The architecture of MobileNetV3 block. (d) The architecture of a DenseNet with five layers, with a growth rate of 4; CNN, convolutional neural networks.

The depth of representations is of great importance for many computer visual tasks. In theory, deeper neural networks should get better results at training, but in reality, deeper neural networks are more difficult to train because of degradation problem. Residual Network (ResNet) was proposed by He et al., which introduces a deep residual learning framework to address the degradation problem (He et al., 2016). The core concept of ResNet is shortcut connections which can be done directly by simple identity mapping. This would allow raw input information to be transmitted directly to later layers of the network without additional parameters and computational burden, helping solve the problem of gradient degradation, which was caused by multilayer backpropagation of error signals. ResNet26 model adopts a stack of three layers which are 1 × 1, 3 × 3, and 1 × 1 convolutions kernels, called bottleneck block. The architecture of bottleneck block is shown in Figure 3a.

#### 2.6.4 SE‐ResNet50

Squeeze‐and‐Excitation Residual Network (SE‐ResNet) is another popular architecture, which consists of SE blocks and ResNet bottleneck blocks (Hu et al., 2020). Each 2‐layer block in the 34‐layer ResNet is replaced with this three‐layer bottleneck block, resulting in a 50‐layer ResNet. Then, SE blocks are integrated into the ResNet50 after the nonlinearity following each convolution. SE block that contains squeeze operation and excitation operation conducts attention or gating on the channel. This kind of attention mechanism enables the importance of each feature channel to be automatically acquired through learning, promotes the useful features, and suppresses the features that are not useful to the current task. The schema of SE‐ResNet bottleneck block is illustrated in Figure 3b.

#### 2.6.5 MobileNetV3_large_100

MobileNetV3 is the latest version of MobileNet, published in 2019 as a lightweight network (Andrew et al., 2019). The four core innovations of MobileNetV3 are bneck block which contains the inverted residual with linear bottleneck proposed by MobileNetV2, depthwise separable convolutions proposed in MobileNetV1, SE block which learns to use global information to selectively emphasize informative features and suppress less useful ones, and H‐swish activation function, which is aimed to reduce computation and improve performance, instead of swish function. The MobileNetV3 block is shown in Figure 3c. MobileNetV3 is available in two versions, MobileNetV3_large and MobileNetV3_small. MobileNetV3_large gets higher accuracy on the ImageNet classification task but takes more training time than MobileNet‐V3_small.

#### 2.6.6 DenseNet169

The core idea of densely connected convolutional networks (DenseNet) is dense connectivity, which establishes the connection between different layers. The input of one layer contains the output of the previous layer in addition to the output of all previous layers. This connectivity contributes to enhance transmission of features and utilize features more effectively and mitigate gradient explosion or gradient disappearance. At the same time, concatenating feature maps learned by different layers can help increase the variation in the input of subsequent layers (Krizhevsky et al., 2017). DenseNet performs well despite the lack of training data as the final output of DenseNet can use different levels of features to make decisions. DenseNet model mainly contains dense layer and transition layer. A certain number of dense layers are stacked to form a dense block. A five‐layer dense block is shown in Figure 3d.

### 2.7 Pre‐training and training from scratch

The above six classical CNN models developed on the ImageNet data set were used as pre‐training models applied to the task of predicting concealed AP patients by using sinus rhythm electrocardiogram, and then, the models were fine‐tuned. However, considering that the ECGs were made up of waves and different from the daily life images in ImageNet data set, negative transfer might occur, which would affect the training and development of the model. Therefore, the method of random initialization was also adopted, and the network was trained from scratch.

Resolving the problem of the number of class labels to be predicted at the output of the above classical models differs for our target domain. We employed the common strategy of replacing the “fully connected” and “softmax” layers of the selected networks with 2‐neuron layers of the same types to implement two classifications (normal and concealed AP).

### 2.8 Model hyperparameters

We trained the selected models on ECG training set by adopting random initialization and ImageNet pre‐training, respectively. Each model was trained for 90 epochs. To investigate and compare the performances of the selected models on predicting concealed AP, we adopted common hyperparameters optimized for the models with pre‐training and applied the same hyperparameters to the models with random initialization. The initial learning rate was set to 10^−3^, and the batch size was set to 8. All models accepted the same input ECG images size of 1600*800, Adam Optimizer and categorical cross‐entropy loss function were selected. Meanwhile, cosine annealing was adopted by the learning rate decay.

### 2.9 Statistical analysis

Descriptive statistics were applied to report the clinical characteristics of patients included in this study. Continuous variables were expressed as mean ± standard deviation. Categorical variables were expressed as ratios or percentages. Levene's test was used to check the homogeneity of variance. Normally distributed data were compared using the independent Student's *t*‐test. Chi‐squared test was used for categorical variables. Measures of diagnostic performance included the ROC areas under the curve (AUC), accuracy, sensitivity, specificity, and the F1 score. We used two‐sided 95% CIs to summarize the sample variability in the estimates. SPSS version 19.0 (SPSS Inc) was used for statistical analysis. All tests were performed with a two‐tailed significance level of .05.

## 3 RESULTS

### 3.1 Dataset characteristics

We screened 152 ECG recordings in concealed AP group and 600 ECG recordings in control group according to the inclusion and exclusion criteria. The mean age of patients in control group was 46.0 ± 16.4 years with 49.8% of patients were male. And the mean age of patients in concealed AP group was 46.4 ± 17.7 years with 55.3% of patients were male. There were no statistically significant differences in ECG characteristics between control group and concealed AP group in terms of PR interval and QRS interval. However, the QT interval and QTc were slightly higher in control group than in concealed AP group. The clinical characteristics of these patients are shown in Table 1.

**TABLE 1:** Baseline characteristics of patients and ECGs.

Parameters	Control (*n* = 600)	Concealed AP (*n* = 152)	Total (*n* = 752)	*p* value
Age (years)	46.0 ± 16.4	46.4 ± 17.7	46.0 ± 16.6	.791
Gender (male, %)	299 (49.8)	84 (55.3)	383 (50.9)	.232
Heart rates	78.7 ± 14.5	79.4 ± 14.7	78.9 ± 14.6	.610
PR interval	149.4 ± 18.8	147.5 ± 19.5	149.0 ± 18.9	.287
QRS interval	85.6 ± 11.0	85.8 ± 10.5	85.7 ± 10.9	.865
QT interval	366.0 ± 31.2	354.0 ± 29.1	363.6 ± 31.1	<.001
QTc	414.6 ± 25.0	406.4 ± 25.5	413.0 ± 25.3	<.001

Abbreviations: AP, accessory pathway; ECG, electrocardiography.

### 3.2 Model screening performance

Considering the imbalance of dataset, we selected proper sensitivity and specificity for comparison. The maximal sensitivity was chosen with the cutoff threshold of specificity above 98.0% for each model. The data of the F1‐score, classification accuracy, sensitivity, specificity, and precision rates are shown in Table 2. ResNet26, SE‐ResNet50, MobileNetV3_large_100, and DenseNet169 all could predict concealed AP through normal sinus rhythmic ECGs, while Alexnet and VGG19 models were not appropriate models under this hyperparameter configuration for this task. By comparing the architecture of these four models, they all had shortcut connections or skip connections which connected the output of the front layer/block with the input of the rear layer/block. These connections ensured the information flow between all layers and alleviated the problem of gradient disappearance effectively.

**TABLE 2:** Comparison of evaluation metrics of six classical CNN models

Model	F1 score	Accuracy	Sensitivity	Specificity	Precision
Alexnet (P)	0.328	0.836	0.196	1.000	1.000
Alexnet (R)	0.000	0.796	0.000	1.000	NAN
VGG19 (P)	0.000	0.796	0.000	1.000	NAN
VGG19 (R)	0.000	0.796	0.000	1.000	NAN
Resnet26 (P)	0.899	0.960	0.870	0.983	0.930
Resnet26 (R)	0.909	0.965	0.870	0.989	0.952
SEResnet50 (P)	0.899	0.960	0.870	0.983	0.930
SEResnet50 (R)	0.911	0.965	**0.891**	0.983	0.932
MobilenetV3_large (P)	0.911	0.965	**0.891**	0.983	0.932
MobilenetV3_large (R)	0.921	0.969	**0.891**	0.989	0.953
Densenet169 (P)	0.899	0.960	0.870	0.983	0.930
Densenet169 (R)	**0.932**	**0.973**	**0.891**	**0.994**	**0.976**

Abbreviations: ECG, electrocardiography; NAN, not a number; P, pre‐trained initialization; R, random initialization.

The values in bold represented the optimal observed values shown in Table 2. And DenseNet169 trained with random initialization slightly outperformed other models in terms of the F1‐score, classification accuracy, sensitivity, specificity, and precision, suggesting that it was the most effective model for the target classification task and the target data sample as DenseNet model adopted more intensive connection. In addition, as shown in Table 2, the performance of CNN models with random initialization was not worse than their corresponding pre‐trained initialization.

### 3.3 Receiver operator curves (ROCs)

**FIGURE 4:**
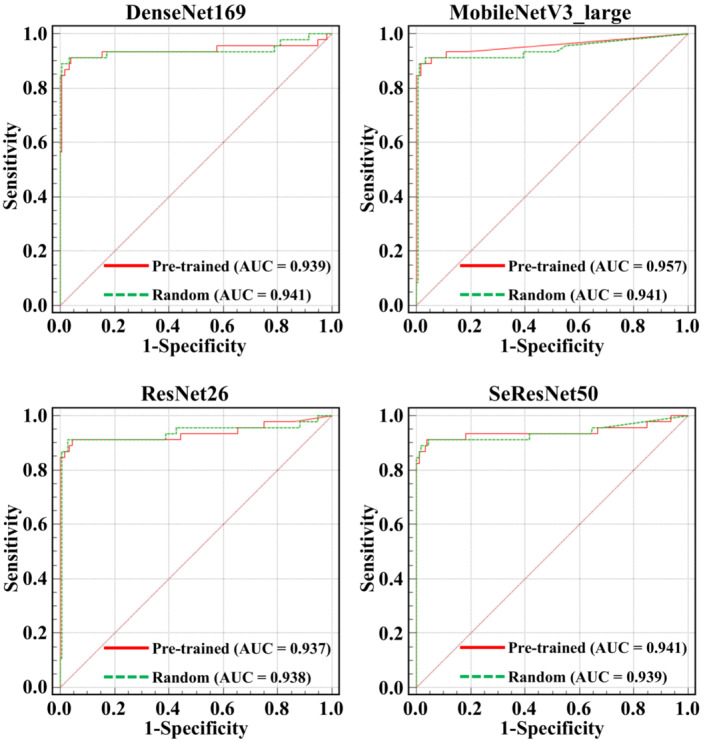
ROCs of the screening performance on testing set of the CNN models between the random initialization and pre‐trained initialization. AUC, area under the curve; ROC, receiver operating characteristic curve; CNN, convolutional neural networks.

To further evaluate the classification performance of these models between the random initialization and pre‐trained initialization, the ROCs of four models were created and shown in Figure 4. All models had a similar performance according to the AUCs.

### 3.4 Training loss

**FIGURE 5:**
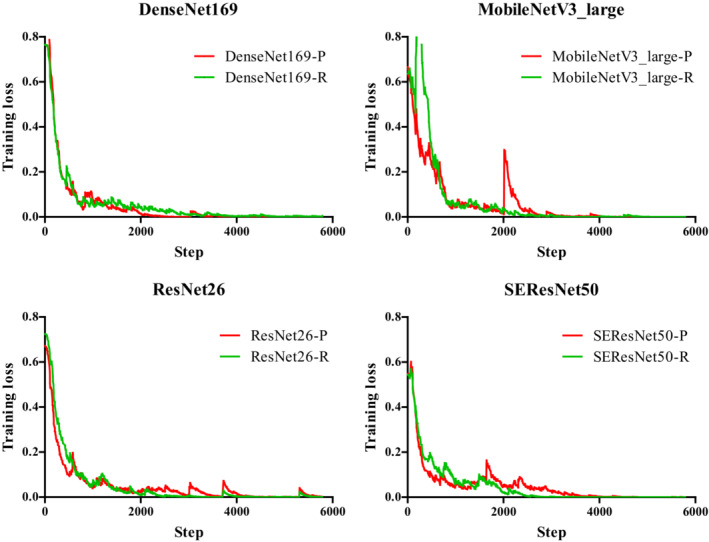
Training loss curves. For each model, the training loss curves were compared between models trained from random initialization and fine‐tuned with ImageNet pre‐trained initialization.

As shown in Figure 5, under the premise of using the common hyperparameters, the above four models could converge completely, indicating that the selected hyperparameters were appropriate. For each model, we compared the training loss curves between models trained from random initialization and fine‐tuned with ImageNet pre‐trained initialization. The models trained from random initialization converged as fast as those initialized from ImageNet pre‐trained initialization, which may challenge our previous cognition of pre‐trained models converged faster. This study showed that models trained from scratch were not inferior to ImageNet pre‐trained models in terms of both model performance and convergence speed of model training on our concealed AP task.

## 4 DISCUSSION

It was known that diagnosis of concealed AP was dependent on electrophysiological study. In the present study, we reported a non‐invasive deep learning method for concealed AP detection from ECG recordings by training six classical CNN models through random initialization and ImageNet pre‐trained initialization, respectively. The main findings of this study were as follows: (1) deep learning with CNNs had significant effects on the automatic extraction of subtle features invisible to naked eyes from ECG recordings, which could assist to diagnose the concealed AP; (2) Models trained on natural images (such as ImageNet) might not be the best models for ECG images, while competitive accuracy of concealed AP prediction was achievable when training on AP dataset from random initialization (“from scratch”) without any pre‐training.

Deep learning is a subtype of machine learning and has shown state‐of‐the‐art performance in medical image. A well‐trained deep learning model could learn patterns within raw ECGs to diagnose sinus rhythm and multiple arrhythmias with compelling performance to cardiologists (Feeny et al., 2020). Attia et al. used deep learning to detect patients with the electrocardiographic signature of atrial fibrillation present during normal sinus rhythm and had practical implications for atrial fibrillation screening (Attia, Noseworthy, et al., 2019). Jo et al. trained a deep learning model to detect PSVT using clinically diagnosed PSVT patients' ECGs during normal sinus rhythm, which demonstrated a high performance in identifying PSVT (Jo et al., 2021). In a previous study, we trained a CNN with normal sinus rhythm ECGs of negative control patients and PSVT procedural patients. This model demonstrated well performance to identify individuals with a high likelihood of PSVT and might have useful implications for PSVT screening and diagnosis (Wang et al., 2022). In the present study, we trained deep learning models with ECGs of healthy patients and concealed AP patients confirmed by electrophysiological study and radiofrequency ablation. Our data demonstrated that ResNet26, SE‐ResNet50, MobileNetV3_large_100, and DenseNet169 but not Alexnet and VGG19 could detect concealed AP through normal sinus rhythmic ECGs. Our results might provide a novel approach to identify concealed AP with ECGs in time that could not be accomplished before. Despite the fact that the early diagnosis is not determined only from an ECG recording, an initial screening of the cases would be useful for the timely application of corresponding treatment, such as medication or surgery to improve life quality of patients.

It is well known that success of deep learning highly depends on the size and quality of dataset, and training a deep learning model often requires a vast quantity of data (Jiang et al., 2020). Large dataset contributes to better performance and generalization ability of the model in general, because it can learn more features from more data. However, larger dataset consumes more labeling cost, needs more powerful computer, and complicated model to extract all the features. In the present study, only 150 ECG images were collected from 421 concealed AP patients. The number of included ECG was relatively small. Small dataset based on high quality also could achieve convincing performance. Ng reported that data quality was more important than data size (Strickland, 2022). Small uniformly distributed, accurately marked, and clean dataset could solve big issues including model efficiency, accuracy, and bias. In some scenarios, there is only a small dataset. Collecting high quality dataset and adjusting model hyperparameters would help us to achieve acceptable performance.

To overcome the problem of limited data size, pre‐trained CNN models with ImageNet (transfer learning) were applied, and models from scratch were trained to compare the performance. Alquran et al. applied transfer learning strategies on pre‐trained AlexNet and GoogleNet to classify five different arrhythmias from ECG waveform, obtaining the highest average accuracy of 97.8% (Alquran et al., 2019). Pal et al. employed the principle of transfer learning for arrhythmia detection and, specifically, used pre‐trained DenseNet, which was trained on ImageNet dataset to classify 29 types of heartbeats and achieved high classification accuracy of 98.92% (Pal et al., 2021). In previous study, pre‐trained CNN could be effective in cross‐modality imaging settings, such as natural images to ECG images (Apostolopoulos & Mpesiana, 2020; Minaee et al., 2020). Therefore, if there is a significant gap between the source pre‐training task and target task, collecting more data, building specific models, and training on the target task from scratch are the solutions worth trying.

In the current literature, CNN models trained from scratch or fine‐tuned from ImageNet models outperformed CNNs that used fixed internal weights (Shin et al., 2016). As the modalities of natural and medical images differed considerably, some researchers questioned the latest medical research preferring ImageNet to medical data, and they demonstrated that medical pre‐training had significant potential (Wen et al., 2021). Considering the particularity of ECG image differs from natural image, we also trained these models from scratch by adopting random initialized weights instead of pre‐trained weights and updated them during training phase. Our study demonstrated that the performance of CNN models with random initialization was not worse than their corresponding pre‐trained initialization. Alzubaidi et al. found that the lightweight model trained from scratch achieved a more competitive performance when compared to the ImageNet pre‐trained model, using three different medical imaging datasets (Alzubaidi et al., 2021). Amit et al. also put forward the point that when using small training samples, training from scratch of domain‐specific deep models (if the size of the model is selected properly) could achieve a superior performance when compared with transfer learning from a network that had been pre‐trained using large training samples in another domain (e.g., natural images; Liu et al., 2019). He et al. report that training from scratch could be no worse than its ImageNet pre‐training counterparts under many circumstances, although ImageNet pre‐training speeded up convergence early in training, but training from random initialization was more robust (He et al., 2018). Therefore, although pre‐trained models have shown an effective performance in several domains of application, pre‐trained models may not offer significant benefits in all instances when dealing with medical imaging scenarios.

### 4.1 Limitations

Current work reflected one of the pilot concealed AP detection study. Enrolling concealed AP patients and collecting preprocedure ECG images was an ongoing process of our project and would allow us to use a more comprehensive dataset for model training, and then got a more reliable prediction accuracy of these models. Besides, the automatic prediction of cases was made using only an ECG recording rather than a more holistic approach based on other factors that might behave at risk factors for the onset of the disease. The exclusion criteria could not rule out all the patients with concealed AP. Maybe patients will suffer from atrioventricular re‐entry tachycardia based on a concealed AP after data collection. Also, future study would focus on locating the waveform which be treated as important features and extracted by the algorithm, which not only solve the problem of black box in deep learning but also make the result more convincing. Some limitations of this study can be overcome in future research.

## 5 CONCLUSION

The present study contributes to the possibility of a low‐cost, rapid, and noninvasive method to diagnose patients with concealed AP. Adopting models trained from random initialization may be also a good choice when dealing with a small medical dataset.

## AUTHOR CONTRIBUTION

The conception and design of the study or analysis and interpretation of data: L.W. and S.D. Acquisition of data: F.Y. and X.J.B. Drafting the article or revising it critically for important intellectual content: L.W. and X.P.B. Final approval of the version to be submitted: S.D., R.X.W., and F.P.

## CONFLICT OF INTEREST STATEMENT

There are no conflicts of interest.

## ETHICS STATEMENT

This study was approved by the ethics committee of the affiliated Wuxi people's hospital of Nanjing medical university.

## Data Availability

Data are available upon request from the authors.
